# Higher global gross primary productivity under future climate with more advanced representations of photosynthesis

**DOI:** 10.1126/sciadv.adh9444

**Published:** 2023-11-17

**Authors:** Jürgen Knauer, Matthias Cuntz, Benjamin Smith, Josep G. Canadell, Belinda E. Medlyn, Alison C. Bennett, Silvia Caldararu, Vanessa Haverd

**Affiliations:** ^1^ Hawkesbury Institute for the Environment, Western Sydney University, Penrith, NSW, Australia.; ^2^ CSIRO Environment, Canberra, ACT, Australia.; ^3^ Université de Lorraine, AgroParisTech, INRAE, UMR Silva, Nancy, France.; ^4^ School of Ecosystem and Forest Science, University of Melbourne, Richmond, VIC, Australia.; ^5^ Botany, School of Natural Sciences, Trinity College Dublin, Dublin, Ireland.; ^6^ iCRAG SFI Research Centre in Applied Geosciences.

## Abstract

Gross primary productivity (GPP) is the key determinant of land carbon uptake, but its representation in terrestrial biosphere models (TBMs) does not reflect our latest physiological understanding. We implemented three empirically well supported but often omitted mechanisms into the TBM CABLE-POP: photosynthetic temperature acclimation, explicit mesophyll conductance, and photosynthetic optimization through redistribution of leaf nitrogen. We used the RCP8.5 climate scenario to conduct factorial model simulations characterizing the individual and combined effects of the three mechanisms on projections of GPP. Simulated global GPP increased more strongly (up to 20% by 2070–2099) in more comprehensive representations of photosynthesis compared to the model lacking the three mechanisms. The experiments revealed non-additive interactions among the mechanisms as combined effects were stronger than the sum of the individual effects. The modeled responses are explained by changes in the photosynthetic sensitivity to temperature and CO_2_ caused by the added mechanisms. Our results suggest that current TBMs underestimate GPP responses to future CO_2_ and climate conditions.

## Teaser

Bringing the latest understanding of plant physiology into models suggests larger increases of future plant productivity.

## INTRODUCTION

Terrestrial vegetation acts as an important mitigator of anthropogenic climate change due to its capacity to take up large amounts of CO_2_ every year through gross primary productivity (GPP), a canopy-scale metric of photosynthetic activity. Terrestrial biosphere models (TBMs) are the main tools to project the spatial and temporal dynamics of GPP and their responses to increases in atmospheric CO_2_, temperature, and other climate change factors. Modeling studies have demonstrated a high sensitivity of GPP simulations and their climate responses to differences in photosynthetic model formulations and assumptions as well as to values of key photosynthetic parameters (*1*–*5*). This has implications for projecting carbon cycling, surface hydrology, energy partitioning, and climate (*6*–*8*) and emphasizes the need for accurate representations of photosynthesis in TBMs.

Current TBMs, however, often do not represent our latest understanding of plant ecophysiological functioning. It is well known that plant responses to environmental cues are plastic and that photosynthesis is not only a function of instantaneous environmental conditions but also dependent on longer-term (days to years) changes of their surroundings (*9*–*11*). Similarly, TBMs still commonly assume an infinite mesophyll conductance (*g*_m_), ignoring clear evidence that *g*_m_ is finite and that CO_2_ concentration available for photosynthesis is lower than usually assumed (*12*, *13*).

Despite the fact that these mechanisms are well established in the literature, they are often not considered within TBMs or only implemented incrementally, which means that our current assessments of climate change impacts do not take our latest physiological understanding into account. One potential explanation for this lack of development is that parameters in photosynthetic models are usually adjusted to observations and therefore can compensate existing structural model errors (*1*, *14*), which allows a satisfactory performance of relatively simple models under present-day conditions. However, such model deficiencies may compromise the predictive capability of these models when confronted with new conditions as imposed by climate change (*15*), an issue that is likely alleviated by more realistic representations of photosynthesis that mirror our latest understanding of plant physiological functioning.

One major barrier for the implementation and parameterization of new mechanisms into TBMs has long been limited data availability across plant functional types (PFTs). However, the availability of relevant physiological data has increased substantially over recent years. For example, Kumarathunge *et al.* (*16*) analyzed a compilation of measurements of photosynthesis performed under different temperatures to infer temperature acclimation and adaptation formulations for tree species around the world, updating an earlier analysis (*9*). Another leaf dataset presented by Maire *et al.* (*17*) confirmed the “coordination hypothesis,” which states that leaf nitrogen (N) is distributed in a way that leads to an approximately equal contribution of Rubisco and RuBP-limited photosynthesis rates (*18*). Knauer *et al.* (*19*) compiled a database of mesophyll conductance (*g*_m_) values across PFTs that can be used for parameterizing *g*_m_ formulations in TBMs.

Scientific efforts increasingly strive to increase model realism through, e.g., plant trait plasticity and more explicitly resolved processes (*20*–*23*), and studies have implemented acclimation of photosynthesis and leaf respiration to growth temperature (*8*, *24*–*28*), an explicit formulation of *g*_m_ (*15*, *19*, *29*), and photosynthetic optimization approaches (*23*, *30*–*32*). These optimization approaches may be implemented by redistributing photosynthetic N to maximize photosynthesis (*30*, *31*), which often predicts a better balance between photosynthetic processes as outlined in the coordination hypothesis (*18*).

Some of these studies found notable increases in the simulated response of GPP to atmospheric CO_2_ concentration [e.g., (*15*, *33*)], and almost all studies found changes in the spatial patterns of GPP [e.g., (*19*, *26*)]. The sound empirical foundation of the mechanisms outlined above suggests that their incorporation into TBMs increases model realism and consequently confidence in projections of future GPP. However, the effects of individual mechanisms on projections of GPP are difficult to assess since the used models, simulation protocols, as well as the reported metrics differ across studies. In addition, new mechanisms are in most cases implemented in isolation, precluding any insights into potential interactions among them.

Here, we incorporated three photosynthetic mechanisms not previously combined in one model framework—photosynthetic acclimation to temperature, explicit *g*_m_, and photosynthetic optimization—into the Community Atmosphere–Biosphere Land Exchange–Populations Orders Physiology (CABLE-POP) TBM (*31*). CABLE-POP contributes to the annual global carbon budget assessment and showed above-average model performance when evaluated against benchmarking datasets (*34*). We conducted global factorial simulations forced by historical (1900–2005) and projected climate (2006–2099) using the RCP2.6 and RCP8.5 climate scenarios. The main goal was to investigate the individual as well as combined effects of the added mechanisms on simulations of GPP. Last, we explored the underlying physiological mechanisms that caused these responses across latitudinal bands.

## RESULTS

### Global GPP simulations into the future

**Fig. 1.**
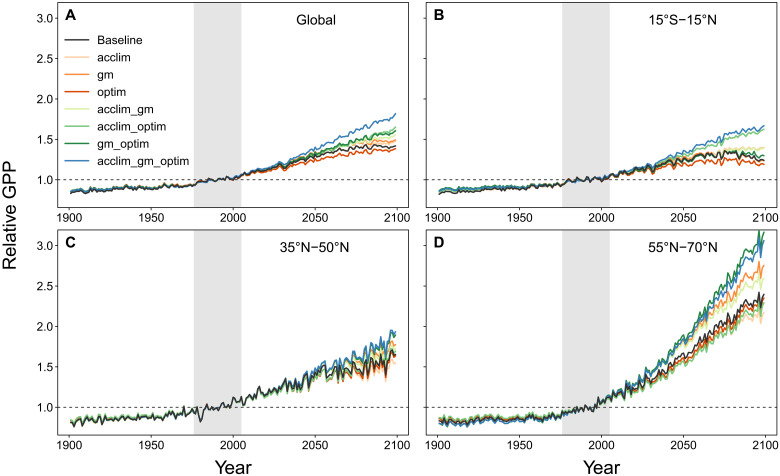
Time series of normalized GPP. Shown are factorial model runs performed in this study for the RCP8.5 climate scenario for (**A**) the globe, (**B**) the inner tropics (15°S to 15°N), (**C**) the temperate zone (35°N to 50°N), and (**D**) the boreal zone (55°N to 70°N). GPP was normalized to the mean of the 1976–2005 reference period (gray shaded area) for each model experiment and each latitude band.

We performed factorial simulations using model versions that excluded or included photosynthetic temperature acclimation (acclim), explicit mesophyll conductance (gm), and photosynthetic optimization (optim), as well as all possible combinations (acclim_gm, acclim_optim, gm_optim, and acclim_gm_optim), resulting in eight experiments (table S1). The experiment that did not consider any of the mechanisms is hereafter referred to as the baseline model. For each simulation, we aggregated annual GPP by calculating the grid cell area–weighted sum globally as well as for three latitude bands representing the inner tropics (15°S to 15°N), the temperate zone (35°N to 50°N), and the boreal zone (55°N to 70°N). We then normalized the aggregated GPP to the mean of the 1976–2005 reference period to compare simulations with different absolute GPP values. Projected future GPP responses diverged widely across model experiments globally as well as in the individual latitude bands (Fig. 1). Throughout zones, more comprehensive representations of photosynthesis generally simulated stronger responses of GPP to anticipated future changes in climate. With all three added mechanisms enabled (acclim_gm_optim; table S1), the model always projected one of the highest increases in GPP, whereas the baseline model was always at the lower end of the predicted GPP range. Experiments with two mechanisms included (green lines in Fig. 1) generally predicted higher GPP responses compared to those that only considered one mechanism (orange/red lines). Note that we focused our analysis on changes in GPP relative to the reference period as opposed to absolute GPP values. In the optim experiment, for example, absolute GPP values were higher compared to the baseline (fig. S1), but its relative increase over the simulation period was less pronounced compared to the baseline and other experiments.

At the global scale, increases in GPP from 1976–2005 to 2070–2099 ranged from 35% in the optim experiment to 68% in the acclim_gm_optim experiment for the RCP8.5 scenario. The simulations further revealed high differences in the GPP response across experiments, and the acclim_gm_optim experiment projected a 20% stronger increase in global GPP compared to the baseline model at the end of the 21st century (2070–2099) relative to the reference period. This corresponded to a simulated global GPP that is, on average, 53.4 Pg C year^−1^ (2070–2099) higher in the acclim_gm_optim experiment compared to the baseline model (but note the offset in the reference period; fig. S1). It is worth noting that the simulations start to diverge not sooner than 2040–2050, under a substantially warmer and higher CO_2_ environment according to the RCP8.5 scenario. An additional set of simulations with the more moderate RCP2.6 climate scenario showed much lower increases in GPP in the first half of the 21st century and a decline thereafter (fig. S2). Despite these contrasting trajectories, differences across experiments were qualitatively similar to those in the RCP8.5 scenario. All results hereafter refer to the RCP8.5 scenario, unless stated otherwise.

### Contribution of individual mechanisms and their interactions

The factorial design allowed us to characterize the contribution of the individual mechanisms to the GPP responses. Temperature acclimation (acclim) and explicit *g*_m_ (gm) led to contrasting GPP responses across latitudes. While the gm run showed the strongest response in the boreal zone and negligible effects in the inner tropics, the opposite pattern was found for photosynthetic temperature acclimation, which had the strongest positive effect in the tropics and a slightly negative effect in the boreal zone (Fig. 1, B and D). The optimization (optim) experiment resulted in consistently lower increases of GPP compared to the baseline model in all latitude bands.

**Fig. 2.**
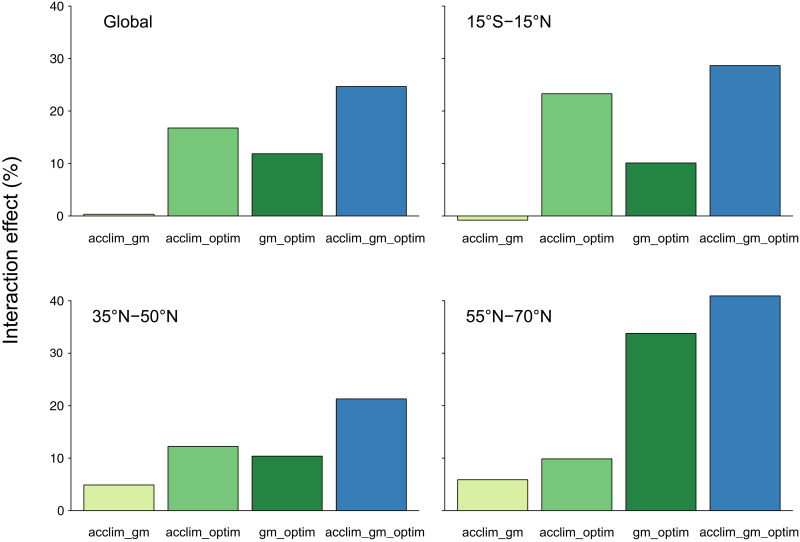
Interaction effects of the mechanisms. Interaction effects were calculated as the difference between the combined effect, calculated from Eq. 6 for the experiment with two or three mechanisms included, and the sum of the effects of the model experiments including the respective mechanisms individually. Values higher than 0 indicate that the combined mechanisms led to stronger increases in GPP than one would expect from the sum of their individual effects.

Our simulations further revealed that the combined effects of the mechanisms on GPP were non-additive, which means that the effects of two mechanisms combined did not equal the sum of the respective individual effects. This was particularly the case in the acclim_optim and gm_optim simulations (Fig. 2). The acclim_optim experiment showed a much stronger response globally and in the tropics than one would expect from the acclim and optim runs alone, which both showed minor effects of opposite direction (Fig. 1, A and B). However, these non-additive effects differed in strength across zones, and the acclim_optim run was relatively close to additive behavior in the boreal zone (Figs. 1D and Fig. 2). The gm_optim experiment displayed a stronger-than-expected increase in the boreal zone and to a lesser extent in the other zones. The acclim_gm_optim experiment showed the strongest interaction effects among mechanisms globally (25%) as well as in all zones (Fig. 2).

To test the robustness of our results with respect to the choice of the baseline model and the assumptions made therein, we performed additional simulations with alternative model formulations as well as a climate scenario from a different Earth system model (see Materials and Methods). These simulations showed consistent effects of the mechanisms across latitudinal bands as well as globally, suggesting that our results are robust to alternative model configurations and assumptions (figs. S3 to S6).

### Global spatial patterns of the effects

**Fig. 3.**
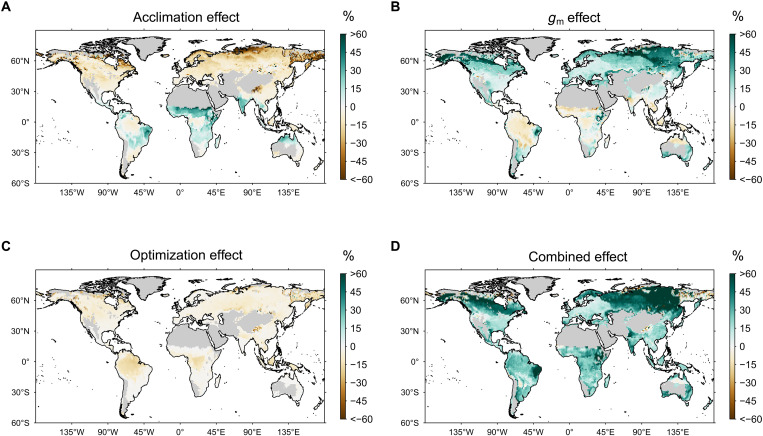
Spatial patterns of the effects of the mechanisms. (**A**) Temperature acclimation of photosynthesis, (**B**) explicit mesophyll conductance (*g*_m_), (**C**) photosynthetic optimization, and (**D**) all mechanisms combined. Effects were calculated according to Eq. 6 (see Materials and Methods) for the reference (1976–2005) and future time periods (2070–2099) for the RCP8.5 climate scenario.

We next compared the spatial distribution of the relative increases in GPP from the reference period (1976–2005) to the end of the 21st century between the model versions including each mechanism individually or combined and the baseline model version (Eq. 6) (Fig. 3). The effects of temperature acclimation (the acclim experiment) on the increase in GPP shows a pronounced latitudinal gradient with the strongest positive effects in the subtropics, near-neutral effects in the inner tropics, and negative effects in the extra-tropics (Fig. 3A). Contrarily, the gm run showed the strongest (positive) effects in the boreal zones and slight negative effects in most parts of the tropics. Effects of the photosynthetic optimization routine (the optim run) were consistently negative, i.e., increases in GPP over the 21st century were weaker compared to the baseline version, the magnitude of which is slightly stronger in the inner tropics compared to other regions. Notably, the combined effect (all three mechanisms included; Fig. 3D) revealed positive effects in almost all regions of the globe, again demonstrating strong non-additive effects among the mechanisms. For example, the Amazon basin showed negative effects for all three mechanisms individually but a positive effect in the combined run. The combined effect was highest in the boreal zone and lowest in the temperate regions, with humid and subhumid regions in the tropics showing intermediate effects. Tundra regions showed no clear patterns and small absolute GPP.

### Underlying causes of the diverging GPP responses

**Fig. 4.**
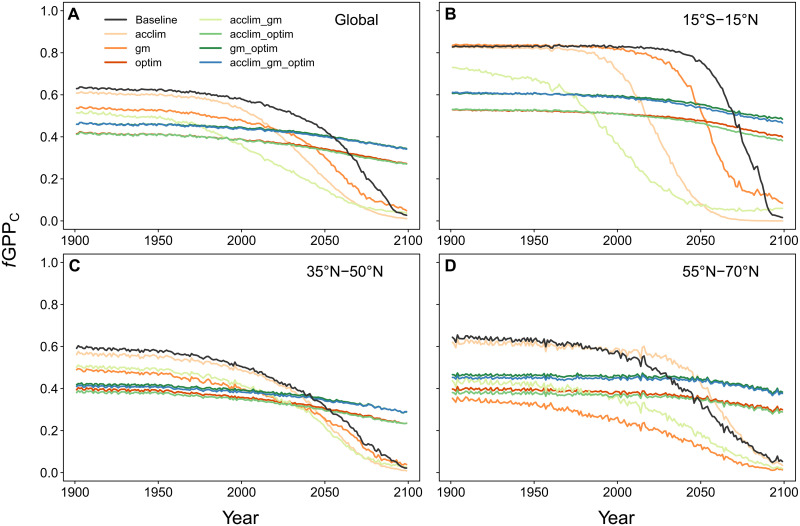
Time series of the fraction of GPP limited by Rubisco activity (*f*GPP_C_) in C_3_ vegetation. (**A**) Globally and for three latitudinal bands (**B** to **D**) in the RCP8.5 climate scenario.

We investigated the sources for the contrasting GPP responses displayed in Figs. 1 and 3. We found that the effects of the three added mechanisms can be explained to a large extent by changes in the temperature and CO_2_ sensitivity of photosynthesis. In C_3_ plants, all three mechanisms cause changes in *b*_JV_ (fig. S7), the ratio between the maximum electron transport rate (*J*_max_), and the maximum carboxylation rate (*V*_cmax_) in the model developed by Farquhar *et al.* (*35*). The value of *b*_JV_ directly affects the fraction of photosynthesis limited by Rubisco activity, henceforth referred to as *f*GPP_C_. *f*GPP_C_ is an important indicator for the CO_2_ sensitivity of photosynthesis because Rubisco-limited photosynthesis (with *V*_cmax_ as the main parameter) is more responsive to atmospheric CO_2_ concentration compared to RuBP regeneration–limited photosynthesis (with *J*_max_ as the main parameter) (*36*). Figure 4 reveals two types of *f*GPP_C_ responses to future climate among the eight model runs: experiments including optim show a relatively constant value around 0.5 (0.4 to 0.6) for all experiments with only a slight decrease over the 21st century. This behavior reflects the capability of the optim model version to redistribute N between Rubisco- and RuBP regeneration–limited processes. Contrarily, *f*GPP_C_ decreases strongly in runs without optim, the shape of which was largely experiment and region specific (Fig. 4). By accounting for the fact that *f*GPP_C_ is a strong determinant of photosynthetic CO_2_ sensitivity, one would expect all experiments including optim to show a stronger response of GPP to elevated CO_2_ concentrations in the future. However, this was only partly the case, and changes in *f*GPP_C_ alone are not sufficient to explain the behavior across experiments, for example, why the optim run shows a lower GPP response compared to other experiments despite its high *f*GPP_C_ at the end of the 21st century.

**Fig. 5.**
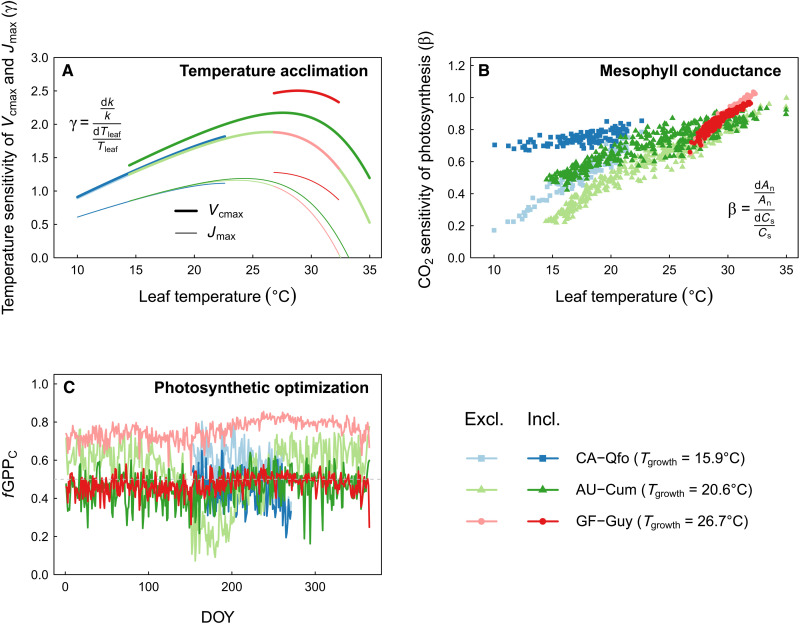
Main direct effects of the introduced mechanisms on photosynthetic behavior. (**A**) Instantaneous temperature sensitivity of *V*_cmax_ and *J*_max_ (elasticity γ) in dependence of leaf temperature with and without photosynthetic acclimation to temperature, (**B**) instantaneous CO_2_ sensitivity of net photosynthesis (elasticity β) in dependence of leaf temperature with and without an explicit *g*_m_, and (**C**) the fraction of GPP limited by Rubisco activity (*f*GPP_C_) with and without photosynthetic optimization for current climate. The faint and full colors denote exclusion (excl.) and inclusion (incl.) of the mechanism, respectively. In all cases, excl. refers to the baseline model, and incl. refers to the model version with the respective mechanism included but the other two mechanisms excluded. Data points represent mean daily variables within the growing season across the simulation period. *k* = *V*_cmax_ or *J*_max_, *T*_leaf_ = leaf temperature, *A*_n_ = net photosynthesis, *C*_s_ = CO_2_ concentration at the leaf surface, *T*_growth_ = average growth temperature in the growing season. Colors denote the three sites (table S4) from the FLUXNET 2015 dataset: CA-Qfo (boreal forest), AU-Cum, (warm-temperate evergreen forest), and GF-Guy (tropical rainforest).

It is important to note that temperature acclimation and *g*_m_ altered not only *b*_JV_ but also the instantaneous photosynthetic temperature and CO_2_ sensitivity, respectively. To explore these effects, we compared simulations with and without the corresponding mechanism for three sites located in contrasting eco-climatic settings (Fig. 5). The temperature acclimation routine increased the temperature sensitivity (elasticity γ, Eq. 7) of *J*_max_ and *V*_cmax_, with a stronger effect for *V*_cmax_ and under higher leaf temperatures (Fig. 5A). The explicit *g*_m_ model version (gm) increased the instantaneous photosynthetic CO_2_ sensitivity (elasticity β, Eq. 8) under low leaf temperatures but had minor or slightly negative effects under high temperatures (Fig. 5B). The optimization routine solely caused changes in *b*_JV_ and thus *f*GPP_C_ for all sites under most conditions (Fig. 5C) but left instantaneous sensitivities unchanged.

Both changes in *f*GPP_C_ (Fig. 4) and in the instantaneous sensitivities (Fig. 5) are needed to explain the results seen in Figs. 1 and 3. Increases in the instantaneous CO_2_ (gm) and temperature (acclim) sensitivities explain why the acclim_optim and gm_optim runs show a higher simulated GPP response in the future than the optim run, despite having a similar *f*GPP_C_. The combination of *f*GPP_C_ and instantaneous sensitivities also explains the non-additive effects seen in the results (Figs 1 and 2): for example, the strong response of acclim_optim run in the inner tropics (Fig. 1B) compared to the acclim or optim runs is the result of a higher *f*GPP_C_ in combination with a higher γ (Fig. 5A), which is particularly pronounced under warm temperatures.

## DISCUSSION

In this study, we assessed the implications of accounting for three photosynthetic mechanisms on projections of global GPP under climate change. The three mechanisms—photosynthetic acclimation to temperature, explicit mesophyll conductance, and photosynthetic optimization—are strongly supported by leaf-level observations but are currently either ignored or only partly considered in TBMs. Our simulations demonstrate that more advanced models of photosynthesis that account for these mechanisms tend to predict higher GPP responses to climate change conditions compared to more basic representations that do not or only partially account for them.

A higher-than-expected increase of GPP under future climate change would have important implications for global carbon cycle assessments as GPP is the main pathway for atmospheric CO_2_ entering the terrestrial biosphere. In TBM projections, a higher GPP is generally associated with an increase in the terrestrial carbon sink, a higher positive carbon-concentration feedback and a less negative carbon-climate feedback (i.e., smaller decrease in global net land carbon uptake for a given increase in temperature), given existing constraints of, e.g., water and nutrient availability (*37*–*39*). A higher vegetation productivity also increases leaf area growth, canopy conductance, and transpiration, affecting surface energy partitioning, land surface temperature, and associated interactions with the atmosphere (*40*–*42*), with likely mitigating effects on climate change globally (*43*).

Global GPP is poorly constrained by observations, but several lines of evidence point to a higher-than-expected increase of GPP over the last decades (*44*). A strong global GPP increase has been detected in atmospheric carbonyl sulfide concentration observations (*45*), the seasonal amplitude of atmospheric CO_2_ (*46*), as well as in eddy covariance data (*47*), although inconsistencies across approaches remain [e.g., (*48*)]. In general, these observations suggest that most TBMs underestimate the increase in GPP over the last decades (*33*), which gives confidence in our simulations here.

Results from our one-factor-only experiments (acclim, gm, and optim) were consistent with previous studies. Photosynthetic acclimation to temperature had more positive (or less negative) effects on the GPP response to climate projections in the tropics compared to higher latitudes, which was also the case in previous implementations using other TBMs [(*26*, *27*, *41*); but note the different metrics used to describe the effects]. *g*_m_ was found to be the most important factor in the boreal zone but had lower effects in the tropics, also consistent with previous studies (*15*, *19*). In both cases, effects can be primarily attributed to changes in the instantaneous temperature (acclim) and CO_2_ sensitivities (gm) of photosynthesis and their variation with leaf temperature, which explains differences in its effects across climate zones and latitudinal bands. For example, an explicit *g*_m_ increased photosynthetic CO_2_ sensitivity more strongly under cold than under warm temperatures, which led to the described stronger responses in the boreal zone compared to the tropics (*15*, *19*). The implementation of the optimization approach led to the same behavior as shown by Ali *et al.* (*30*), as both studies found a weaker increase of an optimized GPP to future climate compared to the baseline model. The reason for this is a change in *b*_JV_ arising from the optimization algorithm, which again affects *f*GPP_C_ and thus the CO_2_ sensitivity of photosynthesis. Our optimization approach is identical to the implementation as presented by Haverd *et al.* (*33*) but uses a different baseline model (see Materials and Methods), which explains the discrepancy between the effect found here (weaker GPP response compared to the baseline model) and in (*33*) (higher GPP response).

Our model simulations revealed strong interactions among the implemented mechanisms as the effects of experiments considering two or three mechanisms were higher than the summed effects of the experiments considering the same mechanisms individually. These findings further demonstrate why the implementation of only one of the mechanisms may not fully capture the response of GPP to rising CO_2_ concentrations and temperatures. For example, the optim version simulated a more realistic *f*GPP_C_ through adjustments of *b*_JV_ (*17*), but it did not affect instantaneous responses (biochemical parameters in the temperature response functions of *V*_cmax_ and *J*_max_). In contrast, the acclim version altered fast responses of *V*_cmax_ and *J*_max_ but may not capture long-term changes in *b*_JV_ under future higher CO_2_ concentrations. The model version combining both mechanisms (acclim_optim) simulated stronger GPP responses as it accounted for both short- and long-term changes in the parameters. Notably, none of the three mechanisms alone could reproduce the GPP response that emerged from the acclim_gm_optim run in any of the latitude bands, emphasizing the need for more comprehensive implementations of photosynthesis in global models.

An inevitable side effect of adding new mechanisms to models is that new uncertainties are introduced or stated differently, previously disregarded components of uncertainty are made explicit. We acknowledge that these need to be identified and addressed to ensure that the predictive capability is maintained with more complex model structures. Key uncertainties in the representation of temperature acclimation in TBMs include the question to what extent observed decreases in *b*_JV_ with temperature are caused by decreases in *V*_cmax_ or increases in *J*_max_, both of which could result in the same *b*_JV_ (*26*). This aspect affects the value of *f*GPP_C_ and thus photosynthetic CO_2_ sensitivity in the model but could not yet be fully resolved by a recent data analysis (*16*). An additional uncertainty revolves around thermal limits of acclimation. In this study, the assumption was made that temperature acclimation occurs even if temperatures fall outside the temperature range observed in the field (*16*). While the same assumption was made in other analyses (*26*, *27*), some studies imposed a temperature limit on acclimation responses (*25*, *41*). Determining limits to temperature acclimation under long-term warming should be a priority in climate change research. Our findings further suggest that accounting for temperature acclimation in C_4_ plants has strong positive effects on projections of GPP in lower latitudes. Temperature acclimation in C_4_ plants has so far not been considered in TBMs, and we note that the formulation used here is yet to be consolidated by more comprehensive data compilations that allow defining temperature acclimation responses of individual parameters in C_4_ photosynthesis models.

The largest uncertainties associated with *g*_m_ lie in the characterization of the *g*_m_ response to environmental factors, including soil moisture and temperature. In particular, the strength of the temperature response of *g*_m_ has strong effects on the GPP response (fig. S8). The temperature response function determined by Bernacchi *et al.* (*49*) as used here has been confirmed by two other independent studies over a wide temperature range (*50*, *51*) but was in all cases measured in the tropical species *Nicotiana tabacum* (tobacco), which may not adequately represent plant types across the globe. Assuming that the more moderate response as measured by Walker *et al.* (*51*) for the temperate species *Arabidopsis thaliana* is more representative of temperate or boreal species would lead to a weaker response of *g*_m_-including runs in these regions but still lead to a stronger GPP response to an anticipated future climate (fig. S8). The implementation of an adequate temperature response in TBMs is further complicated by the fact that robust patterns across species or climate zones could not be identified (*52*) and are yet to emerge from the literature.

Optimization approaches are generally more parsimonious and generalizable compared to other approaches (*11*), but their predictive capability critically depends on the validity of the underlying assumptions. While photosynthesis in C_3_ plants has been shown to be coordinated in data representing ambient conditions (*17*), studies using high CO_2_ experiments suggest that the shift in *b*_JV_ is not as large as predicted from optimization (*53*, *54*). The extent to which photosynthetic coordination and associated parameters such as *b*_JV_ hold under elevated CO_2_ and temperature conditions in C_3_ plants (*55*) deserve further investigations. In C_4_ plants, photosynthetic optimization approaches would need to consider not only the relationship between *J*_max_ and *V*_cmax_ but also how these relate to the maximum phosphoenolpyruvate carboxylation rate (*V*_pmax_) (*56*). To represent optimization in TBMs for C_4_ plants, future studies will need to assess to what extent measured photosynthetic parameters are coordinated in this plant group.

In summary, our results point to (i) stronger increases of future GPP with more comprehensive representations of photosynthesis in TBMs; (ii) non-additive effects among the mechanisms, i.e., different model behavior when mechanisms are implemented in isolation or in combination; (iii) contrasting effects of individual mechanisms on GPP across latitude bands and regions but positive effects in all parts of the globe when all mechanisms are combined; and (iv) changes in photosynthetic CO_2_ and temperature sensitivity as the main explanatory factor for the observed differences. Last, the fact that the different model experiments began to diverge only under a considerably warmer and higher CO_2_ environment emphasizes the need to evaluate model formulations particularly for these conditions. Hence, experiments and data analyses focusing on physiological and biochemical measurements from plants growing under high CO_2_ concentrations (>550 ppm) and temperatures (>+2°C) will be crucial for future-proofing the representation of plant physiology in TBMs.

## MATERIALS AND METHODS

### CABLE-POP model description

All simulations were performed with the TBM CABLE-POP (*31*) revision 8768, which consists of a biogeophysics module (*57*), a biogeochemistry module including a N cycle (*58*), and woody demographics (*59*). CABLE-POP simulates a one-layered, two-leaf (sunlit and shaded) canopy. Leaf-level physiological parameters are scaled to the canopy level separately for sunlit and shaded fractions of the canopy depending on their LAI fractions and a canopy N extinction coefficient [see (*57*) for details]. Stomatal conductance (*g*_s_) is simulated according to Medlyn *et al.* (*60*) with a PFT-specific minimum *g*_s_ as described by Lombardozzi *et al.* (*61*). Equations for C_3_ and C_4_ photosynthesis were implemented according to Farquhar *et al.* (*35*) and Collatz *et al.* (*62*), respectively. N availability has immediate effects on plant physiology through an implemented dependency of *V*_cmax25_ on leaf N content as derived by Walker *et al.* (*63*). The model further accounts for temperature acclimation of leaf respiration following the formulations presented by Atkin *et al.* (*64*), which were also applied to maintenance respiration of stems and roots.

### Photosynthetic mechanisms added to CABLE-POP

#### Temperature acclimation of photosynthesis

In the baseline version of CABLE-POP, the temperature response of *V*_cmax_ and *J*_max_ is independent of growth temperature and described by a modified Arrhenius function

$$k_{T_{\mathrm{leaf}}}=k_{25}\;\exp\left(\frac{H_{a}(T_{\mathrm{leaf}}-T_{\mathrm{ref}})}{T_{\mathrm{ref}}RT_{\mathrm{leaf}}}\right)\frac{1+\exp\left(\frac{T_{\mathrm{ref}}\text{Δ}S-H_{d}}{T_{\mathrm{ref}}R}\right)}{1+\exp\left(\frac{T_{\mathrm{leaf}}\text{Δ}S-H_{d}}{T_{\mathrm{leaf}}R}\right)}$$ (1)

where *k*_*T*leaf_ is either *V*_cmax_ (mol m^−2^ s^−1^) or *J*_max_ (mol m^−2^ s^−1^) at leaf temperature *T*_leaf_ (K), *k*_25_ is *V*_cmax_ or *J*_max_ at the reference temperature *T*_ref_ (298.15 K = 25°C), *H*_a_ is the activation energy (J mol^−1^), ∆*S* is the entropy term (J mol^−1^ K^−1^), *H*_d_ is the deactivation energy (J mol^−1^), and *R* is the universal gas constant (8.314 J mol^−1^ K^−1^).

In the presence of photosynthetic acclimation to temperature, *H*_a_ and ∆*S* in Eq. 1 are functions of growth temperature (*T*_growth_), defined as the mean air temperature of the preceding 31 days, and/or the temperature of origin (*T*_home_), here defined as the mean air temperature of the warmest month over the preceding 20 years. *H*_d_ in Eq. 1 was fixed at 200 kJ mol^−1^ as in the study of Kumarathunge *et al.* (*16*). The functions used here were derived by Kumarathunge *et al.* (*16*) for a variety of woody species grown under a wide range of *T*_growth_ and *T*_home_. The general form of the equation is

$$P_{x}=a+bT_{\mathrm{growth}}+cT_{\mathrm{home}}+d(T_{\mathrm{growth}}-T_{\mathrm{home}})$$ (2)

where *P_x_* represents either *H*_a_ or ∆*S* for both *V*_cmax_ and *J*_max_. Values of parameters *a*, *b*, *c*, and *d* can be found in table S2. *P_x_* shows acclimation to *T*_growth_ if *b* ≠ 0 and both acclimation to *T*_growth_ and adaptation to *T*_home_ if *c* ≠ 0 and *d* ≠ 0. With acclimation turned on, the ratio of *J*_max_ to *V*_cmax_ at 25°C (*b*_JV_) also changes with *T*_growth_ and *T*_home_ according to Eq. 2. We assumed that changes in *b*_JV_ along with changes in *T*_growth_ or *T*_home_ are entirely caused by *J*_max25_, whereas *V*_cmax25_ stays constant. The acclimation formulations for *V*_cmax_ and *J*_max_ implicitly include possible acclimation effects on *g*_m_ (*16*); hence, we did not apply temperature acclimation to *g*_m_.

We derived formulations for temperature acclimation of *V*_cmax_ in C_4_ plants through a constrained fitting procedure in which parameters *a* and *b* in Eq. 2 were determined such that the slope between *T*_opt_ and *T*_growth_ corresponded to the one presented by Yamori *et al.* (*10*) with the parameters *H*_a_, *H*_d_, and ∆*S* corresponding to the formulation without acclimation at a growth temperature of 20°C (table S3). We further assumed a negligible effect of *T*_home_ on *H*_a_ and ∆*S* as was found for C_3_ plants, i.e., *c* = *d* = 0 (*16*). The fitted parameters can be found in table S2.

No thermal limits to temperature acclimation were implemented here. That means that Eq. 2 was applied irrespective of the value of *T*_growth_ or *T*_home_. In the experiments without temperature acclimation, values of *H*_a_, ∆S, and *b*_JV_ were set to those given by Eq. 2 with a *T*_growth_ of 15°C and a *T*_home_ of 25°C for C_3_ plants [values close to the global mean of *T*_growth_ data presented by Kumarathunge *et al.* (*16*)] and a *T*_growth_ of 20°C for C_4_ plants.

#### Mesophyll conductance

Mesophyll conductance (*g*_m_) represents the conductance to CO_2_ transfer within plant leaves (*65*) and is usually only implicitly considered in TBMs. Here, an explicit representation of *g*_m_ was implemented similarly as described by Knauer *et al.* (*19*). Mesophyll conductance at the canopy level (*G*_m_) responds to environmental factors as follows

$$G_{m}=g_{m25}\times \text{Π}\times {fw}_{\mathrm{soil}}\times {fT}_{\mathrm{leaf}}$$ (3)

where *g*_m25_ is unstressed mesophyll conductance at 25°C at the leaf level that varies across PFTs (*19*), and Π is the canopy integration factor that scales leaf-level *g*_m_ to its canopy-level analog in the same way as other photosynthetic parameters (*57*). Π is calculated separately for shade and sunlit canopy fractions. *fw*_soil_ is a soil water stress factor that depends on available soil moisture in the root zone and ranges from 0.15 to 1 (*66*, *67*). A minimum value of 0.15 was implemented to avoid *G*_m_ falling to 0. *fT*_leaf_ is the temperature response function of *g*_m_ as in Eq. 1 with *k*_25_ replaced by *g*_m25_ and parameter values as given in table S3 (*15*, *68*).

Since *fT*_leaf_ is a key uncertainty for the effects of *g*_m_ on projections of photosynthesis (*68*), two different formulations were implemented (*49*, *51*). Both formulations represent a modified Arrhenius function (Eq. 1) but differ in their parameter values (table S3) as they were derived for two species originating from contrasting climate zones: the (sub)tropical species *N. tabacum* in the study of Bernacchi *et al.* (*49*) and the temperate species *A. thaliana* in the study of Walker *et al.* (*51*). The two functions may thus represent the uncertainty of GPP simulations associated with the temperature response of *g*_m_ across climate zones.

An explicit *g*_m_ does not affect existing formulations of *g*_s_ and photosynthesis in the model. However, a finite *g*_m_ reduces the simulated CO_2_ concentration available for photosynthesis from the intercellular CO_2_ concentration (*C*_i_) to the chloroplastic CO_2_ concentration (*C*_c_), which requires adjustment of photosynthetic parameters to not bias calculations of photosynthesis (*15*, *68*). Here, the photosynthetic parameters *J*_max25_ and *V*_cmax25_, as well as their ratio *b*_JV_, were adjusted from a *C*_i_-based to a *C*_c_-based representation within the photosynthesis module using simulated *A*_n_-*C*_i_ curves as described in full detail by Knauer *et al.* (*19*). Since *g*_m25_ is correlated with *V*_cmax25_ defined on a *C*_i_ basis (*V*_cmax25,Ci_), which varies with leaf N content in CABLE-POP (*63*), *g*_m25_ also varies along with *V*_cmax25,Ci_

$$g_{m25}=g_{m25,\mathrm{ref}}+s_{\mathrm{gv}}\;(V_{\mathrm{cmax}25,\mathrm{Ci}}-V_{\mathrm{cmax}25,\mathrm{Ci},\mathrm{ref}})$$ (4)

where *g*_m25,ref_ and *V*_cmax25,Ci,ref_ are the *g*_m__25_ and *V*_cmax25,Ci_ under average leaf nutrient contents, respectively, and *s*_gv_ is the slope between *g*_m25_ and *V*_cmax25,Ci_ (table S3) extracted from the dataset presented by Knauer *et al.* (*69*).

#### Photosynthetic optimization

The photosynthetic optimization approach was implemented as described in full detail by Haverd *et al.* (*31*, *33*). In short, the routine calculates *b*_JV_ such that canopy net photosynthesis is maximized over a certain time period (here 5 days) given the N content available for photosynthetic processes. The latter is represented as an effective photosynthetic N content at canopy level (*N*_eff_) and given by

$$N_{\mathrm{eff}}=V_{\mathrm{cmax}25}+N_{\mathrm{costJV}}\times b_{\mathrm{JV}}\times V_{\mathrm{cmax}25}$$ (5)

where *N*_costJV_ represents the relative N cost of photosynthesis associated with RuBP regeneration–limited processes relative to those associated with Rubisco-limited processes. Following Haverd *et al.* (*31*), *N*_costJV_ is assumed to equal 2.0 (with *J*_max25_ expressed in units of μmol (electrons) m^−2^ s^−1^ and assuming a requirement of 4 mole of electrons per mole CO_2_), but other values have also been reported in the literature (*53*, *70*).

The optimization algorithm represents a redistribution of photosynthetic N between Rubisco- and RuBP regeneration–limited processes, which leads to an approximately equal contribution of the two limitation states to canopy photosynthesis under ambient conditions (*31*). Since an explicit representation of *g*_m_ alters *b*_JV_, *N*_costJV_ also needs to be adjusted accordingly and was recalculated as *N*_costJV_ = *b*_JV,Ci_/*b*_JV,Cc_. If both temperature acclimation and optimization were implemented, then *b*_JV_ was not adjusted through acclimation (Eq. 2) but through the optimization routine. In that case, temperature acclimation affected all parameters listed in table S2 except for *b*_JV_. We did not implement optimization for C_4_ plants since we are not aware of any studies that provide evidence for photosynthetic optimization in C_4_ plants.

Note that our optimization approach is identical to the implementation as presented by Haverd *et al.* (*33*) except that *b*_JV_ was assumed to be higher in the baseline model. We assumed a *b*_JV_ of ca. 1.8 here following Kumarathunge *et al.* (*16*), whereas Haverd *et al.* (*33*) assumed a value of 1.3. The higher *b*_JV_ leads to a higher fraction of GPP limited by Rubisco activity (*f*GPP_C_) and thus to a higher CO_2_ sensitivity in the baseline model presented here compared to the baseline model presented by Haverd *et al.* (*33*).

### Simulation protocol

#### Model experiments

We conducted a full factorial model simulation experiment, in which all possible combinations of the three added mechanisms [explicit *g*_m_ (gm), photosynthetic acclimation to temperature (acclim), and photosynthetic optimization (optim)] were simulated (table S1). This setup resulted in eight separate model experiments and allowed for the calculation of individual and combined effects of the mechanisms on GPP.

#### Global simulations

CABLE-POP was run globally at 1° spatial resolution from 1901 to 2099 using climate forcing from the IPSL-CM5A-LR model as provided by phase 2a of the Inter-Sectoral Impact Model Intercomparison Project (ISI-MIP) (*71*). The forcing provides a seamless transition of climate variables as well as CO_2_ concentrations and N deposition rates from historical to future time periods for different Representative Concentration Pathways (RCPs). For the main simulations, we selected the RCP8.5 scenario which represents a very high emission pathway. Additional simulations were performed using the RCP2.6 scenario, which is consistent with the temperature target of the Paris Agreement. All simulations were performed in offline mode, i.e., CABLE-POP was not coupled to an atmospheric model. Land use change (LUC) was simulated from 1901 to 2018 according to the LUH2 v2h dataset (*72*), and no LUC was simulated past 2018. To test the robustness of our results with respect to alternative climate forcing data, model formulations, and parameters, we performed an additional set of simulations in which we tested the effects of an alternative climate forcing using the HadGEM2-ES climate model (*71*), an alternative parameterization of soil water stress effects on plant physiology (*73*), an alternative stomatal conductance model (*74*), as well as different parameter values for the canopy N extinction coefficient (*57*).

#### Postprocessing

GPP from global simulations was first averaged across the vegetation types present in each grid cell by calculating the area-weighted mean from which the annual mean of the grid cell was calculated. The effect of an individual mechanism was quantified as

$$\mathrm{Effect}=\frac{{\mathrm{GPP}}_{\mathrm{incl},\mathrm{fut}}-{\mathrm{GPP}}_{\mathrm{incl},\mathrm{hist}}}{{\mathrm{GPP}}_{\mathrm{incl},\mathrm{hist}}}-\frac{{\mathrm{GPP}}_{\mathrm{baseline},\mathrm{fut}}-{\mathrm{GPP}}_{\mathrm{baseline},\mathrm{hist}}}{{\mathrm{GPP}}_{\mathrm{baseline},\mathrm{hist}}}$$ (6)

where “incl” denotes that the mechanism of interest was included, and “baseline” denotes the baseline scenario that did not include any of the three mechanisms presented here. “hist” corresponds to the reference period (1976–2005) and “fut” to the last 30 years of the 21st century (2070–2099). Interaction effects among the mechanisms were calculated as the difference between the combined effect, calculated from Eq. 6 for the experiment with two or three mechanisms enabled, and the sum of the effects of the model experiments including the respective mechanisms individually.

#### Site-level simulations

To gain more detailed insights into the underlying mechanisms, site-level simulations were conducted for three ecosystems that differ in their *T*_growth_ as well as their leaf type and phenology. The three sites (see table S4 for location and site characteristics) were a needle-leaf evergreen boreal forest in Canada (CA-Qfo), a warm-temperate evergreen broadleaf forest in South-East Australia (AU-Cum), and a tropical evergreen rainforest in French-Guiana (GF-Guy). Meteorological forcing was taken from flux towers located at the site, whereas CO_2_ concentrations were mean global annual values. LAI was prescribed from remotely sensed products as described by Ukkola *et al.* (*75*). Results shown here represent multiyear daily means over the simulation period of each site (table S4).

We calculated the elasticity of photosynthetic parameters to changes in leaf temperature (γ) as well as the elasticity of net photosynthesis to changes in CO_2_ concentration (β), which are defined as

$$\gamma =\frac{\frac{dk}{k}}{\frac{dT_{\mathrm{leaf}}}{T_{\mathrm{leaf}}}}$$ (7)

$$\beta =\frac{\frac{dA_{n}}{A_{n}}}{\frac{dc_{s}}{c_{s}}}$$ (8)

where *k* is either *V*_cmax_ or *J*_max_, *T*_leaf_ is the leaf temperature, *A*_n_ is the net photosynthesis, and *c*_s_ is the CO_2_ concentration at the leaf surface.
