# Supplementary material for: Higher global gross primary productivity under future climate with more advanced representations of photosynthesis
Source: Sci Adv. 2023 Nov 17;9(46):eadh9444. doi: 10.1126/sciadv.adh9444 (PMC10656065; doi:10.1126/sciadv.adh9444)
Supplement: Supplementary file 1 — Figs. S1 to S8 Tables S1 to S4 [file sciadv.adh9444_sm.pdf]

Science Advances
AAAS

Supplementary Materials for

Higher global gross primary productivity under future climate with more advanced representations of photosynthesis

Jürgen Knauer *et al.*

Corresponding author: Jürgen Knauer, j.knauer@westernsydney.edu.au

Sci. Adv. **9**, eadh9444 (2023)
DOI: 10.1126/sciadv.adh9444

This PDF file includes:

Figs. S1 to S8
Tables S1 to S4

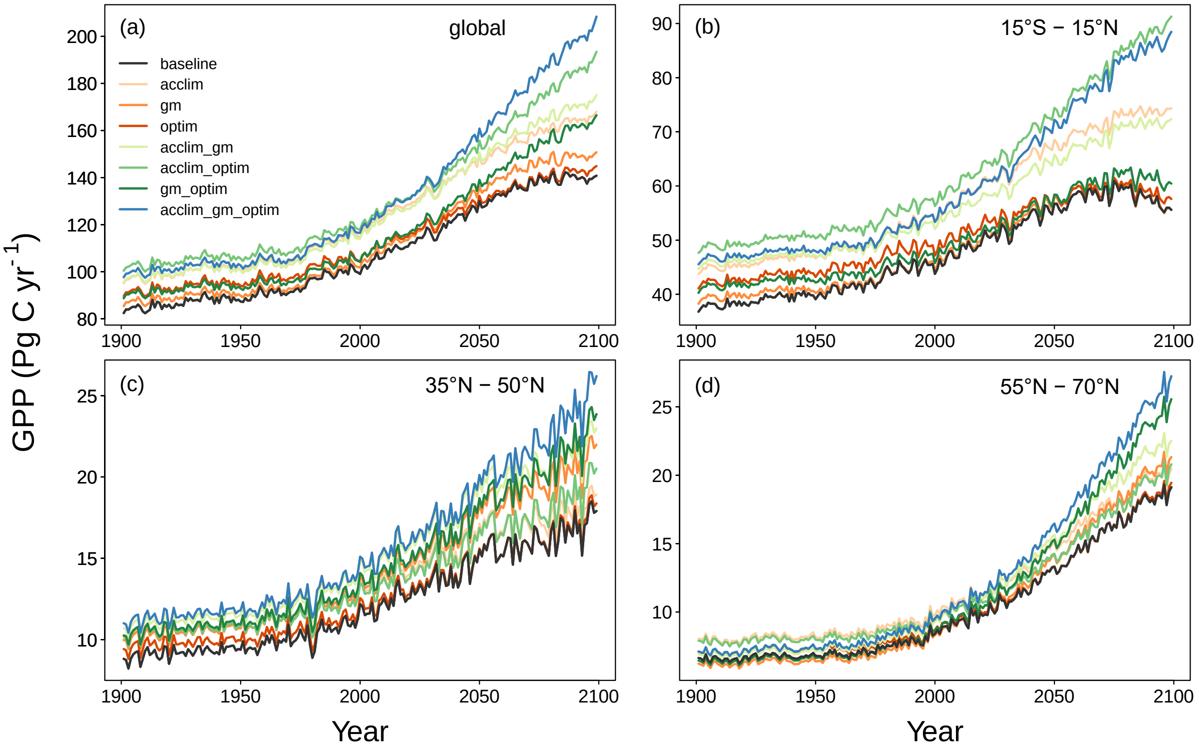

Fig. S1. Time series of absolute gross primary productivity (GPP) globally and for the three latitude bands shown in Figure 1.

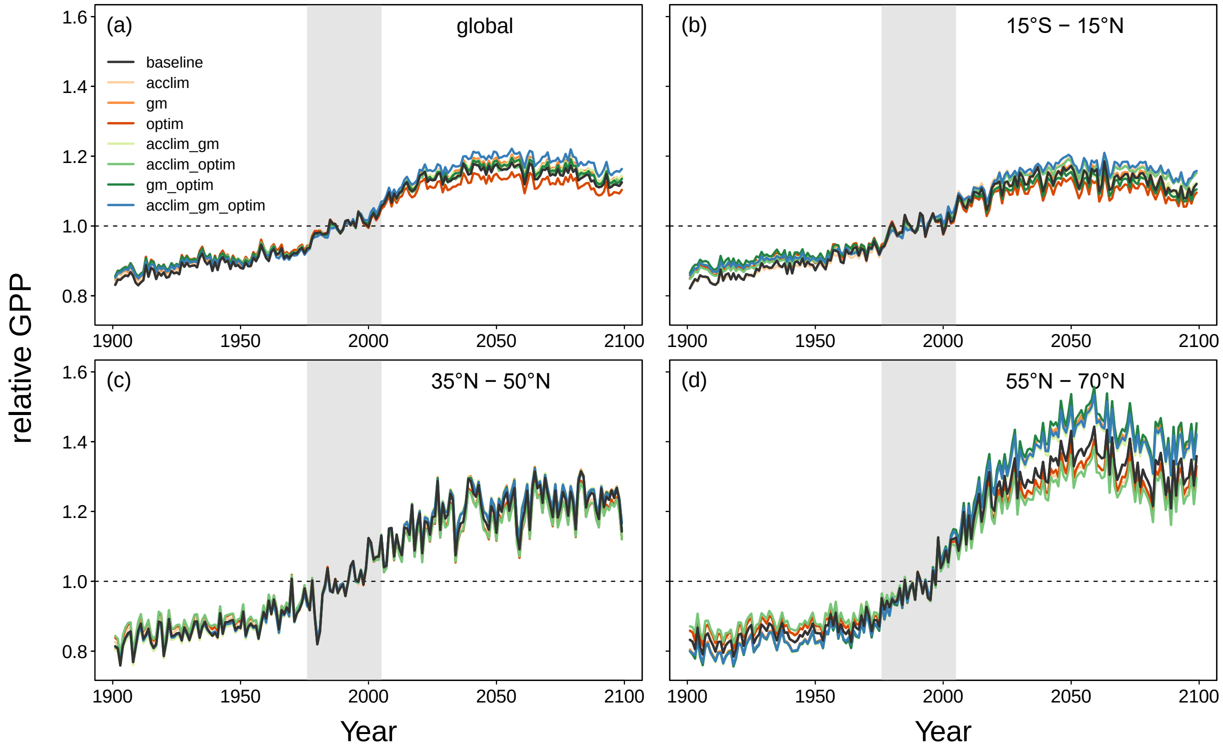

Fig. S2. same as Figure 1, but for the RCP2.6 scenario.

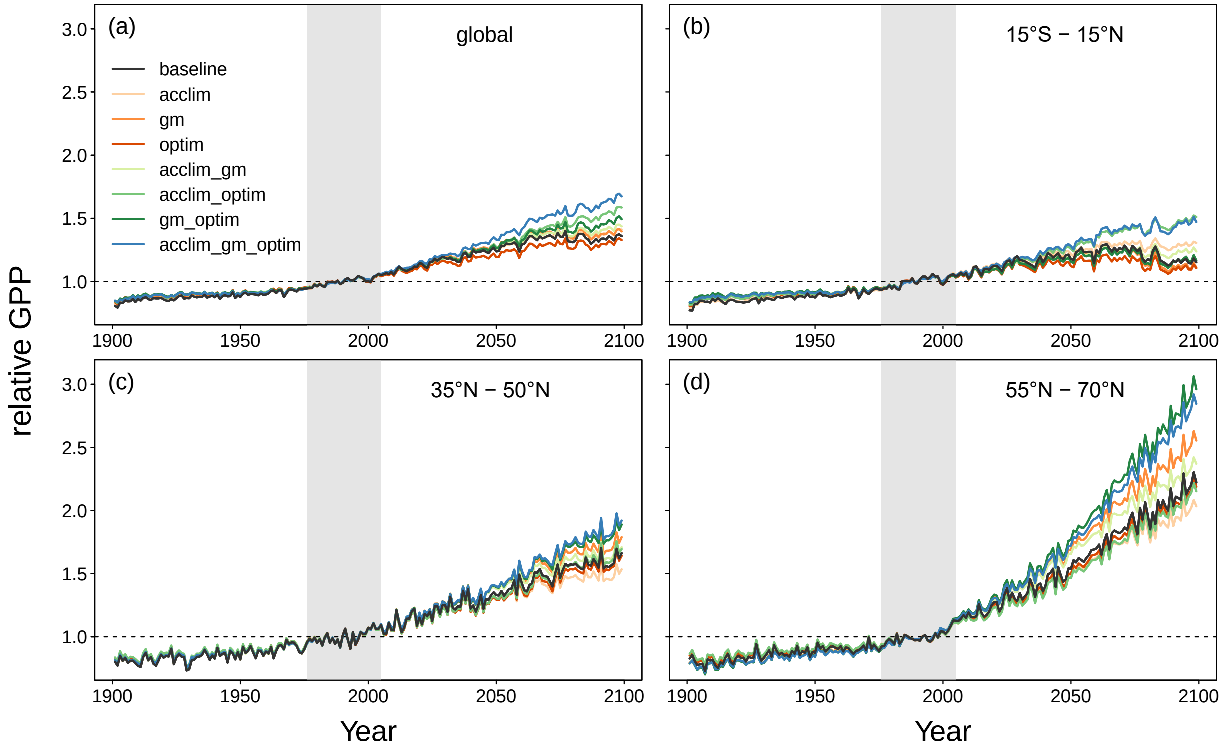

Fig. S3. Same as Figure 1, but with climate forcing (RCP8.5 scenario) from the HadGEM2-ES model (71).

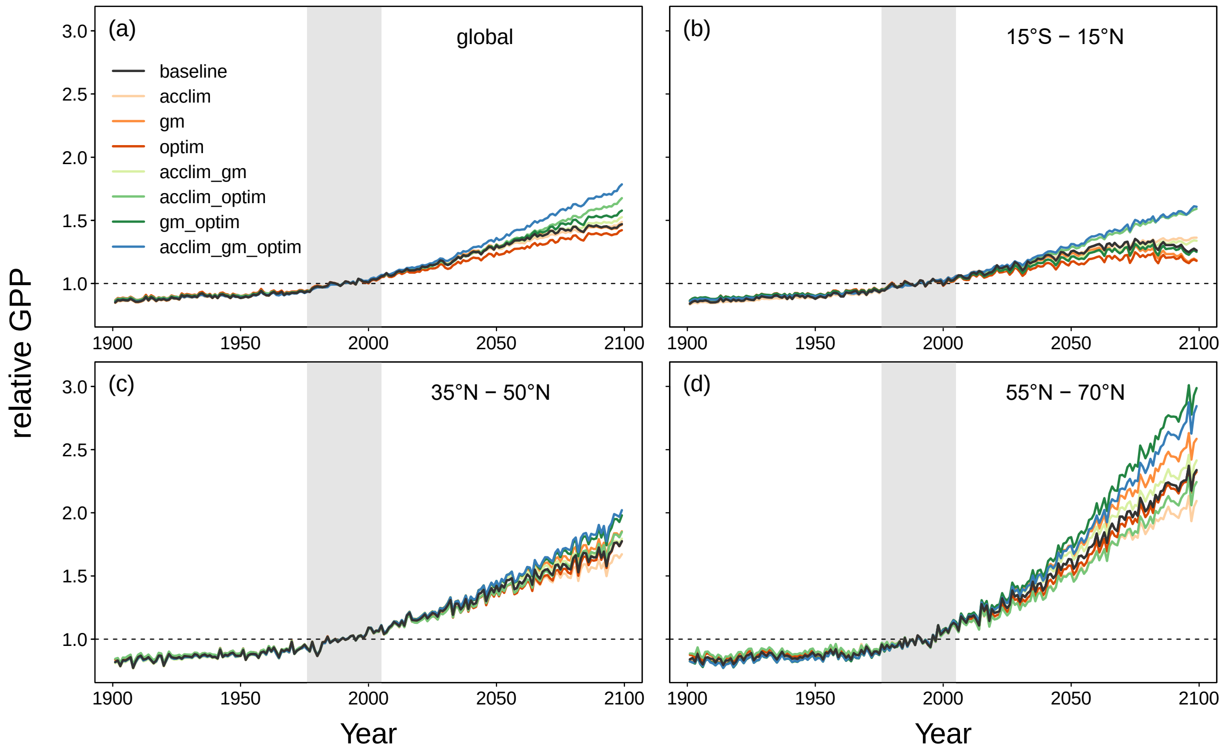

Fig. S4. Same as Figure 1, but with the soil moisture sensitivity function of Haverd et al., 2013 (67) replaced with the one presented in Lai & Katul, 2000 (73).

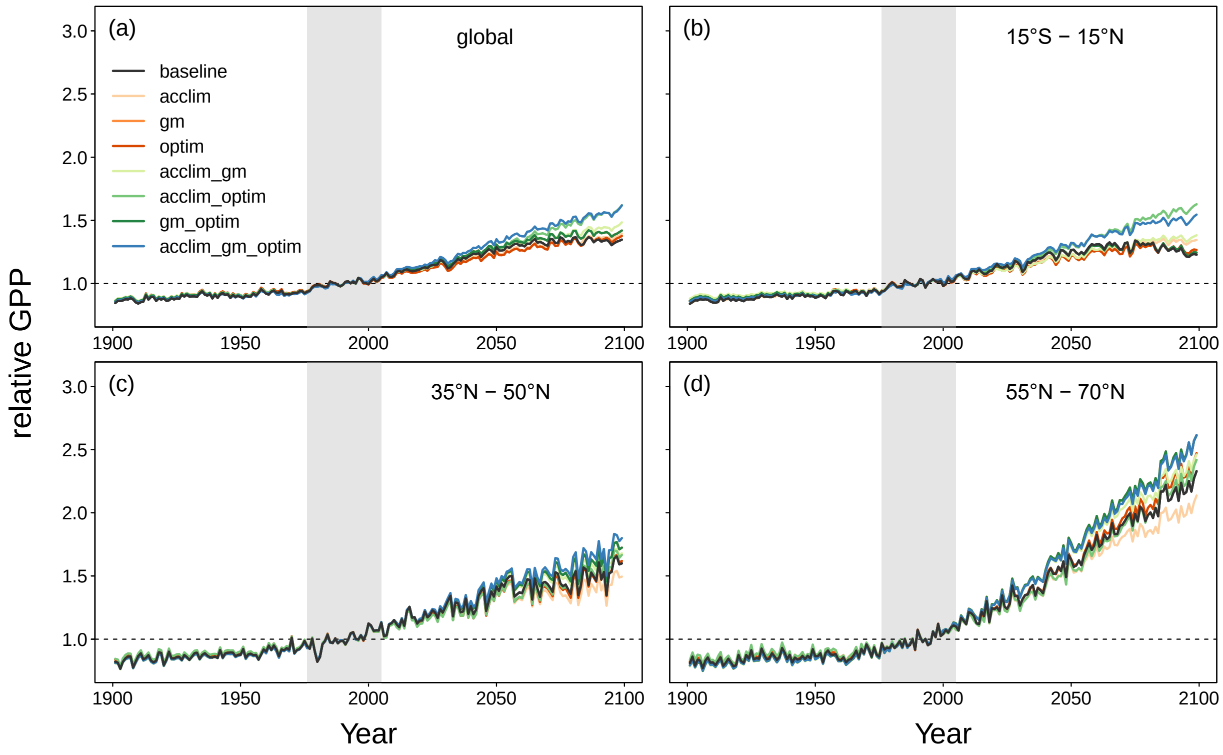

Fig. S5. Same as Figure 1, but with the Medlyn et al., 2011 (60) stomatal conductance model replaced with the one presented in Ball et al., 1987 (74).

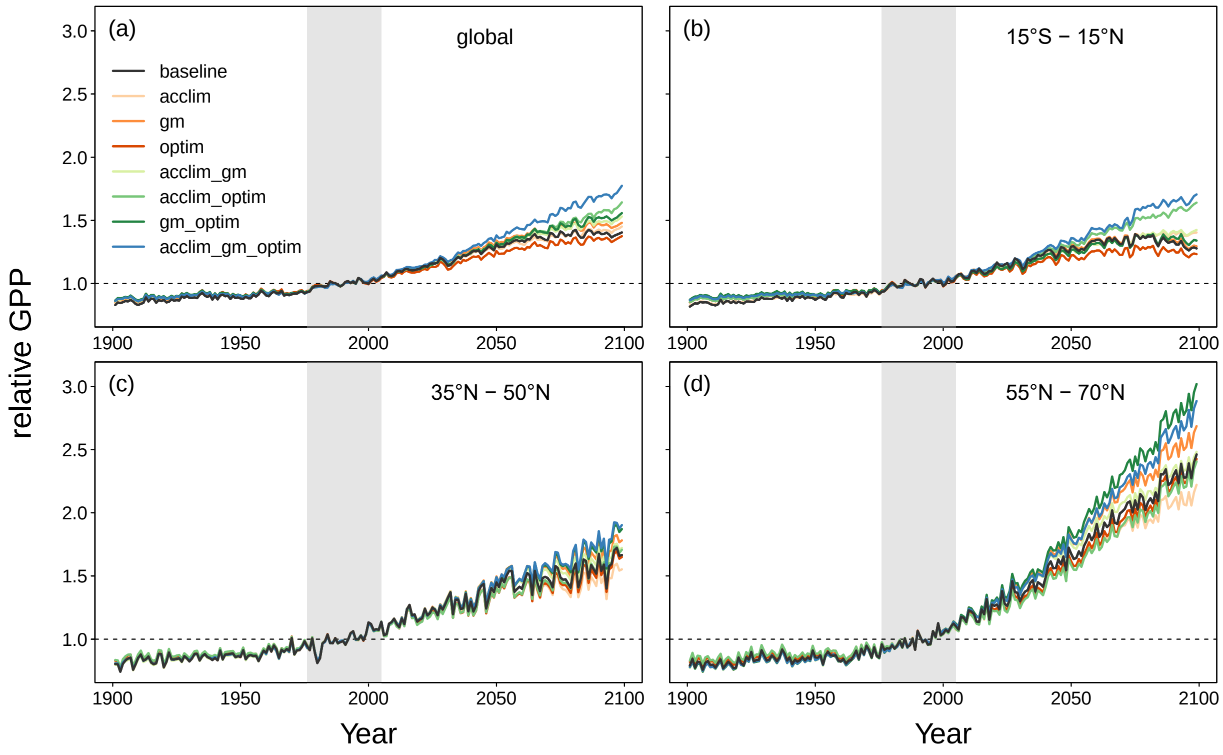

Fig. S6. Same as Figure 1, but with a different value of the within-canopy leaf nitrogen (N) extinction coefficient leading to a shallower distribution of leaf N within the canopy. The following parameters were changed: k_n: -40%; V_{cmax25}: +10%. See Wang & Leuning, 1998 (57) for a detailed description of the parameters.

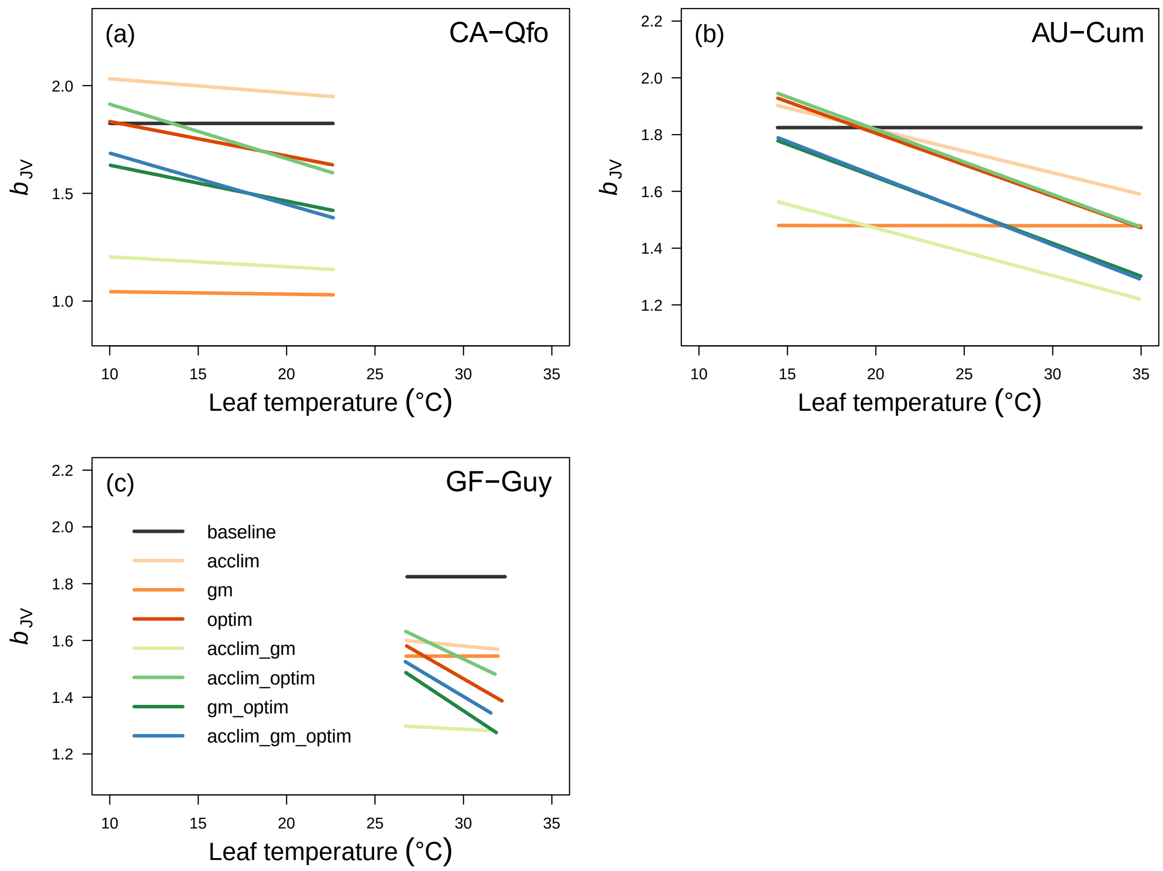

Fig. S7. The J_{max} to V_{cmax} ratio (b_{JV}) simulated by the different model versions for three sites (see Table S4) under ambient atmospheric CO_2 concentrations and climate conditions.

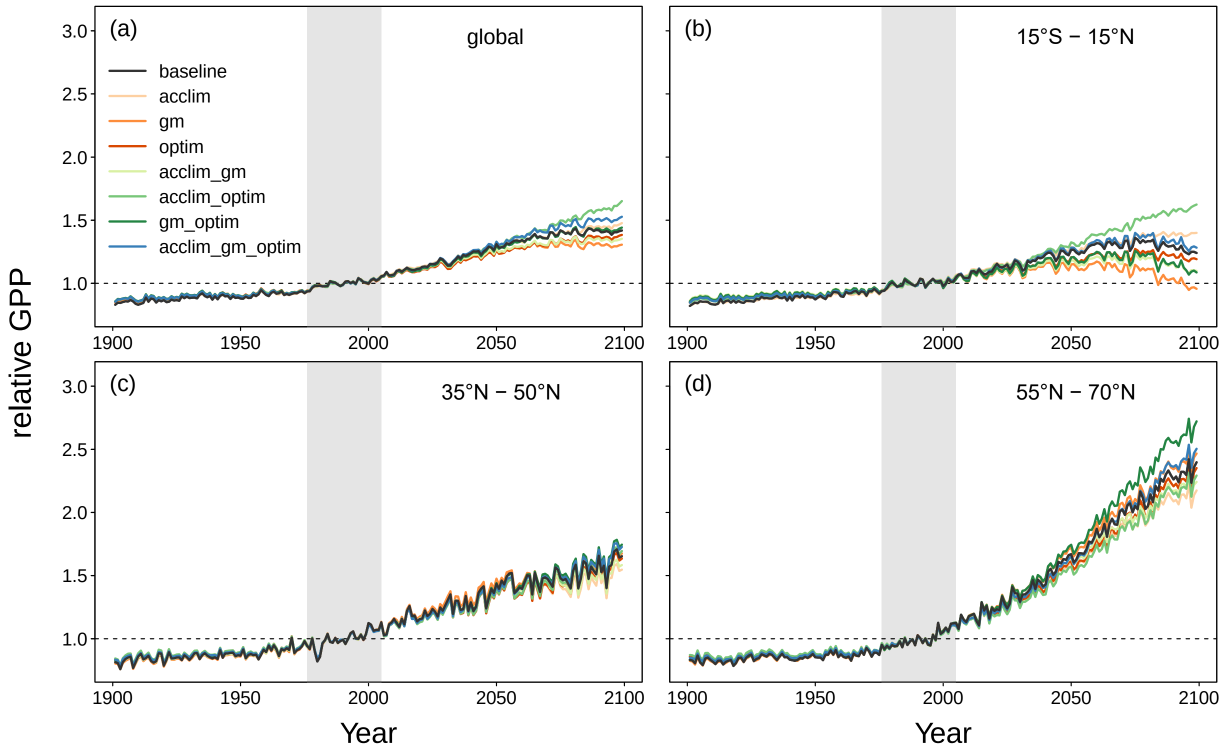

Fig. S8. same as Figure 1 but with the temperature response function of Walker et al., 2013 (51) for the *gm* simulations.

Table S1.

Model experiments conducted in this study and mechanisms included.

Experiment	acclimation	explicit g_m	optimisation
baseline			
acclim	x		
gm		x	
optim			x
acclim_gm	x	x	
acclim_optim	x		x
gm_optim		x	x
acclim_gm_optim	x	x	x

Table S2. Parameter values used in the acclimation function (Eq. 2).

	$H_{av,C3}$	$H_{aj,C3}$	$\Delta S_{v,C3}$	$\Delta S_{j,C3}$	$b_{JV,C3}$	$H_{av,C4}$	$\Delta S_{v,C4}$
a	42.6	40.71	645.13	658.77	2.56	45.0	472.0
b	1.14	0	-0.38	0	0	1.05	-0.1289
c	0	0	0	-0.84	-0.0375	0	0
d	0	0	0	-0.52	-0.0202	0	0

$H_{av,C3}$ = activation energy of V_{cmax} in C_3 plants (kJ mol^{-1}); $H_{aj,C3}$ = activation energy for J_{max} in C_3 plants (kJ mol^{-1}); $\Delta S_{v,C3}$ = entropy term of V_{cmax} in C_3 plants (kJ mol^{-1} K^{-1}); $\Delta S_{j,C3}$ = entropy term of J_{max} in C_3 plants (kJ mol^{-1} K^{-1}); $b_{JV,C3}$ = J_{max} to V_{cmax} ratio at 25°C in C_3 plants; $H_{av,C4}$ = activation energy of V_{cmax} in C_4 plants (kJ mol^{-1}); $\Delta S_{v,C4}$ = entropy term of V_{cmax} in C_4 plants (kJ mol^{-1} K^{-1}).

Table S3. Main photosynthetic parameters used in this study.

Parameter	Description	Value	Unit
$H_{av,C3}$	Activation energy of V_{cmax} in C_3 plants[a]	59.7	kJ mol^{-1}
$H_{dv,C3}$	Deactivation energy of V_{cmax} in C_3 plants[a]	200	kJ mol^{-1}
$\Delta S_{v,C3}$	Entropy term of V_{cmax} in C_3 plants[a]	639.43	J mol^{-1} K^{-1}
$H_{aj,C3}$	Activation energy of J_{max} in C_3 plants[a]	40.71	kJ mol^{-1}
$H_{dj,C3}$	Deactivation energy of J_{max} in C_3 plants[a]	200	kJ mol^{-1}
$\Delta S_{j,C3}$	Entropy term of J_{max} in C_3 plants[a]	642.97	J mol^{-1} K^{-1}
$H_{av,C4}$	Activation energy of V_{cmax} in C_4 plants[a]	66.0	kJ mol^{-1}
$H_{dv,C4}$	Deactivation energy of V_{cmax} in C_4 plants[a]	145	kJ mol^{-1}
$\Delta S_{v,C4}$	Entropy term of V_{cmax} in C_4 plants[a]	469.42	J mol^{-1} K^{-1}
$H_{ag,B2002}$	Activation energy of g_m (ref 49)	49.6	kJ mol^{-1}
$H_{dg,B2002}$	Deactivation energy of g_m (ref 49)	437.4	kJ mol^{-1}
$\Delta S_{g,B2002}$	Entropy term of g_m (ref 49)	1.4	J mol^{-1} K^{-1}
$H_{ag,W2013}$	Activation energy of g_m (ref 51)	7.4	kJ mol^{-1}
$H_{dg,W2013}$	Deactivation energy of g_m (ref 51)	434	kJ mol^{-1}
$\Delta S_{g,W2013}$	Entropy term of g_m (ref 51)	1.4	J mol^{-1} K^{-1}
s_{gv}	Slope between g_m and C_i-based V_{cmax} at 25°C	$0.0035e^{-6}$ [b]<br>$0.0020e^{-6}$ [c]	(mol m^{-2} s^{-1}) / (μmol m^{-2} s^{-1})

[a] in the model versions without photosynthetic acclimation.

[b] for woody needle-leaf trees.

[c] for all other vegetation types.

Table S4. Location and characteristics of the three sites simulated in this study.

Site	Simulation Period	Latitude (°N)	Longitude (°E)	MAT (°C)	MAP (mm)	LAI[a]	PFT
CA-Qfo	2004-2010	49.69	-74.34	-0.4	962	2.8	ENF
AU-Cum	2013-2018	-33.62	150.72	17.7	741	1.4	EBF
GF-Guy	2004-2014	5.28	-52.93	25.7	3041	6.0	EBF

MAT = mean annual temperature; MAP = mean annual precipitation; PFT = plant functional type; EBF = evergreen broadleaf forest, ENF = evergreen needle-leaf forest.

[a] mean growing season leaf area index (LAI).
